# A propensity matched analysis of outcomes and long term survival in stented versus stentless valves

**DOI:** 10.1186/s13019-017-0608-2

**Published:** 2017-05-31

**Authors:** Blake N. Shultz, Tomasz Timek, Alan T. Davis, John Heiser, Edward Murphy, Charles Willekes, Robert Hooker

**Affiliations:** 1 Department of Thoracic and Cardiovascular Surgery, Spectrum Health, Fred and Lena Meijer Heart and Vascular Institute, 100 Michigan St NE, Grand Rapids, MI 49503 USA; 2 Grand Rapids Medical Education Partners, 945 Ottawa Ave NW, Grand Rapids, MI 49503 USA; 3 Department of Surgery, Michigan State University, 15 Michigan St NE, Grand Rapids, MI 49503 USA

**Keywords:** Aortic valve replacement, Stented bioprosthesis, Stentless bioprosthesis, Mortality, Morbidity

## Abstract

**Background:**

To compare the perioperative and long term survival after aortic valve replacement using stentless versus stented valves in a large cohort of patients grouped using propensity score matching.

**Methods:**

From 1991 to 2012, 4,563 patients underwent aortic valve replacement with stentless and stented valves at our institution. Propensity score matching identified 444 pairs using 13 independent variables: incidence of operation, smoking status, renal failure, hypertension, diabetes, peripheral vascular disease, cerebrovascular disease, chronic lung disease, ejection fraction, gender, age, valve status, and use of coronary artery bypass graft. Data were collected from our Society of Thoracic Surgeons database and the Social Security Death Index. Groups were compared using univariate and Kaplan-Meier analysis.

**Results:**

The two groups demonstrated no significant differences for the 13 matching variables and the majority of 30-day outcomes (p > 0.05). The stented valve group showed a higher incidence of postoperative bleeding (3.6% vs 1.1%, *p* = 0.015), but a lower incidence of stroke (0.9% vs. 2.9%, *p* = 0.028). One, five, and 10-year survival was 95.0, 80.7, and 52.8% for stented and 93.2, 80.5, and 51.3% for stentless valves. Overall survival did not differ significantly between the two groups (*p* = 0.641).

**Conclusions:**

Stentless and stented valves had identical 30-day outcomes except for a higher postoperative incidence of bleeding and a lower incidence of stroke in the stented group. There was no significant difference in long term survival between valve types. Both valves may be used for aortic valve replacement with low morbidity and excellent long term survival.

## Background

Aortic valve replacement (AVR) is the standard of care for patients with significant aortic valve pathology [1]. Symptomatic aortic valve disease necessitating replacement may consist of stenosis, regurgitation or a mixed pattern. The stented valve has for decades been the most frequently employed valve in the aortic position when choosing a bioprosthesis. In the 1990’s the stentless aortic valve was introduced in an effort to more closely model the hemodynamics of the native valve, possibly improving valve durability and clinical outcomes [2]. However, modern stented valves offer greatly improved hemodynamic characteristics [3]. Many studies have demonstrated excellent results with both stented [4–7], and stentless valves [3, 8, 9]. Comparative studies have suggested that stented and stentless valves have similar clinical outcomes and hemodynamic profiles such as left ventricular mass (LVM) regression, effective orifice area (EOA), and transvalvular gradients [8, 10–13].

Two studies have shown that stentless valves have improved survival rates and reduced incidence of adverse events [14, 15]. However, no large-scale studies directly comparing the clinical outcomes of these two valves are available in the literature. This study analyzes a single institution’s experience over the past two decades. Stentless and stented valves were compared utilizing propensity matching analysis.

## Methods

### Patients and data source

Between 1991 and 2012, 4,563 patients underwent AVR utilizing a stented or stentless valve. Valves were all implanted according to manufacturer’s recommendations. All patients undergoing aortic valve replacement were prospectively entered into our institution’s Society of Thoracic Surgeons (STS) database. Data collected included patient demographics, comorbidities, operative characteristics, mortality, and 30-day morbidity. This study was approved by the Institutional Review Board (IRB) at Spectrum Health. Individual patient consent was waived.

### Operative technique

Conventional cardiopulmonary bypass was performed utilizing roller head pumps and membrane oxygenators with cold/tepid blood cardioplegia. Stented valves were placed either intra-annular or supra-annular at the discretion of the surgeon using pledgeted sutures. Stented valves included Carpentier-Edwards Perimount (Edwards Life Sciences, Irvine, CA, USA), and Mosaic (Medtronic, Minneapolis, MN, USA). All stentless valves (Medtronic, Minneapolis, MN, USA) were sewn in via a subcoronary technique leaving the noncoronary sinus of the xenograft intact.

### Statistical analysis

Categorical data are expressed as percentages and continuous variables are reported as the mean ± standard deviation (SD). Propensity matched pairs were identified by matching on the following 13 independent variables: incidence of operation, smoking status, renal failure, hypertension, diabetes, peripheral vascular disease, cerebrovascular disease, chronic lung disease, ejection fraction, gender, age, valve status, and whether or not a coronary artery bypass graft was used. Propensity matching of the subjects was performed using the method of Leuven and Sianesi [16]. The propensity matching was performed in three age cohorts: 1998–2002, 2003–2006, and 2007–2010. After matching in each of the three cohorts, the subjects were combined into two matched groups for comparison of the patients who received either the stentless or stented valves. The *χ* ^2^ test and Fisher’s Exact Test were used for the univariate analyses of categorical data and the unpaired *t*-test for was used for the univariate analysis of continuous data. Due to the non-normal distribution of the data, the intensive care unit length of stay (ICU LOS) and hours on mechanical ventilation were log transformed prior to analysis, while the hospital length of stay was transformed using the inverse hyperbolic sine function. A Kaplan-Meier survival curve and a life table were constructed from patient mortality data. Significance was assessed at *p* < 0.05. Stata version 14.2 [StataCorp, College Station, TX, USA] was used to perform the statistical analyses.

## Results

The propensity matched balance between the two groups is shown in Table 1. The stentless valve group was slightly older, while the majority of patients were males in each cohort. Valve pathology was equally distributed with close to 35% in both groups being pure aortic stenosis (AS), with the remainder pure aortic insufficiency (AI) and mixed AS/AI. Seventy-six percent of patients in each group were hypertensive and although 38% of patients were smokers less than 6% in each cohort had moderate or severe lung disease. None of the variables between the two groups were significantly different.

**Table 1:** Preoperative characteristics and their distribution among the patients within the stented group and the stentless group

Variable	Stented [No. (%)] (*n* = 444)	Stentless [No. (%)] (*n* = 444)	*P*-Value
Age (y)^a^	70.9 ± 11.8	71.2 ± 10.8	0.729
Gender (male)	315 (71.0)	318 (71.6)	0.824
Diabetes	122 (27.5)	126 (28.4)	0.765
Smoker	168 (37.8)	167 (37.6)	0.945
NYHA class			0.408
I	20 (6.9)	23 (7.0)	
II	59 (20.3)	77 (23.3)	
III	138 (47.6)	165 (50.0)	
IV	73 (25.2)	65 (19.7)	
Operation Status		0.275	
First operation	391 (88.1)	380 (85.6)	
Reoperation	53 (11.9)	64 (14.4)	
Valve status		0.759	
Insufficient	115 (25.9)	114 (25.7)	
Mixed	159 (38.3)	150 (40.5)	
Stenosis	170 (35.8)	180 (33.8)	
Surgical procedure			0.419
Isolated AVR	195 (43.9)	207 (46.6)	
AVR + CABG	249 (56.1)	237 (53.4)	
Hypertension	336 (75.7)	337 (75.9)	0.938
Cerebrovascular disease	50 (11.3)	56 (12.6)	0.535
Renal Failure	37 (8.3)	39 (8.8)	0.810
Chronic Lung Disease		0.854	
None	392 (88.3)	386 (86.9)	
Mild	32 (7.2)	35 (7.9)	
Moderate	14 (3.2)	14 (3.2)	
Severe	6 (1.4)	9 (2.0)	
Ejection Fraction (%)^a^	53.8 ± 12.9	53.7 ± 12.5	0.930
PVD	52 (11.7)	53 (11.9)	0.917
Year			>0.999
1998–2002	114 (25.7)	114 (25.7)	
2003–2006	70 (15.8)	70 (15.8)	
2007–2010	260 (58.6)	260 (58.6)	

*NYHA* New York Heart Association, *AVR* aortic valve repair, *CABG*, coronary artery bypass graft, *PVD* peripheral vascular disease

^a^Data expressed as Mean ± SD

Table 2 presents the distribution and analyses of 30-day postoperative outcomes within the stented and stentless groups. There were no significant differences found between the groups for postoperative mortality, atrial fibrillation, renal failure or ventilation time (*p* > 0.05). Bleeding that necessitated reoperation was significantly different between the two groups, occurring over three times more frequently in stented patients, compared to stentless patients. Conversely, almost three times as many people in the stentless group had a stroke, compared to the stented valve group (2.9% vs. 0.9%).

**Table 2:** 30-day postoperative outcomes and their distribution among the patients within the sample along with univariate analysis comparing the stentless vs. the stented group

Variable	Stented [No. (%)] (*n* = 444)	Stentless [No. (%)] (*n* = 444)	*P*-Value
30-day mortality	12 (2.7)	13 (2.9)	0.839
Atrial fibrillation	127 (28.6)	136 (30.6)	0.508
MI	12 (2.7)	13 (2.9)	0.839
Stroke	4 (0.9)	13 (2.9)	0.028
ICU admission	155 (34.9)	144 (32.4)	0.435
Readmission within 30 days	38 (8.8)	49 (11.2)	0.227
Ventilation > 48 h	36 (8.1)	47 (10.6)	0.205
Bleeding requiring reoperation	16 (3.6)	5 (1.1)	0.015
Deep sternal infection	2 (0.5)	3 (0.7)	>0.999
Renal failure	25 (5.6)	26 (6.0)	0.885
Renal failure requiring dialysis	14 (3.2)	13 (2.9)	0.845
Length of hospital stay (d)^a^	8.8 ± 7.2	8.8 ± 7.6	0.480
Total ICU Stay (h)^a^	63.7 ± 119.8	71.3 ± 126.0	0.905
Ventilation Time (h)^a^	28.0 ± 118.8	30.9 ± 91.4	0.578

*MI* myocardial infarction, *ICU* intensive care unit, *SD* standard deviation

^a^Data expressed as Mean ± SD

**Fig. 1:**
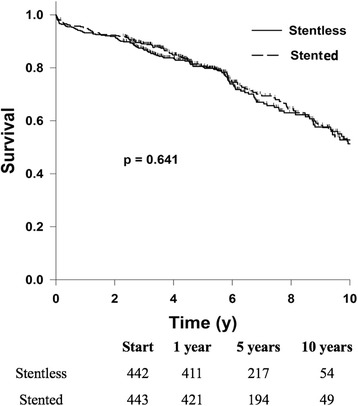
Kaplan- Meier survival curve predicting long term survival of patients within the stented and stentless cohorts. Comparison using the log-rank test gave a *p*-value = 0.641

Table 3 shows survival rated at one, 5 and 10 years after valve implantation. The Kaplan-Meier survival curve is shown in Fig. 1. There is a slight divergence in the curves between 2 and 8 years with survival in the stented slightly better, however by log rank test there is no difference in survival (*p* = 0.64).

**Table 3:** One, five and ten-year survival rates within the stentless and stented groups, based on Kaplan-Meier analysis^a^

Survival	Stentless (*n* = 442) (%)	Stented (*n* = 442) (%)
1 year	93.2 (90.4–95.2)	95.0 (92.6–96.7)
5 years	80.5 (76.3–84.1)	80.7 (76.1–84.4)
10 years	51.3 (43.5–58.5)	52.8 (44.8–60.1)

^a^Data are expressed as % survival, with 95% confidence intervals in parentheses

## Discussion

AVR is a definitive treatment of aortic valve pathology with excellent survival and quality of life in patients. The positive clinical outcomes following AVR result from the relief of the pressure or volume overload on the heart. The choice of stentless or stented valve has been debated as to which most closely mimics the hemodynamics of the natural valve, a characteristic that potentially leads to improved clinical outcome. Prior literature has largely focused on a discussion of the hemodynamic profiles and performance results of each type of valve [4–13, 17]. Borger et al. retrospectively studied 737 patients who underwent AVR with stented and stentless valves [12]. This study found that stentless valves were an independent predictor of LVM regression, and that stentless valves were associated with improved midterm survival by univariate analysis. Smaller, randomized studies and meta-analyses have also compared stentless and stented valves. Most studies have found that stented and stentless valves have comparable clinical outcomes and hemodynamic profiles such as LVM regression, EOA and transvalvular gradients [8, 10–13]. However, alternate studies have suggested that stentless valves offer superior hemodynamic characteristics, although these improvements have not produced significant clinical outcome differences [4–7]. Stentless valves have been shown to produce greater improvement in left ventricular function in patients with a small aortic root or left ventricular impairment [3, 9, 10].

Our study has shown that both stentless and stented valves have excellent short-term survival. We had a 2.7% 30-day mortality in the stented group and a 2.9% mortality in the stentless group which was not statistically significant. Westaby et al. [14] also showed no difference in short-term mortality (before hospital discharge) between stentless and stented valves (8.0% vs 12.1%, *p* = 0.171). However, Luciani et al. [15] showed higher early mortality (before hospital discharge) in stented versus stentless patients (6.2% vs 2.6%, *p* = 0.02).

Long term survival in this cohort was excellent compared to other studies for both types of valves [14, 15]. Westaby et al. [14] showed improved survival in stentless patients at 5 years (84% vs 69%, *p* = 0.023). Luciani et al. showed lower survival at 8 years in stented versus stentless patients (70 ± 5% vs 81 ± 3%, *p* = 0.01) [15]. However, this may be due to bias favoring stented valves in older patients who have higher mortality rates than younger patients. Our propensity matched analysis controlled for some of the age bias by matching patients on age and other preoperative conditions. Our study also included a large number of patients (*n* = 444 of each valve type. Both prior studies had smaller cohort sizes relative to our study. Westaby et al. analyzed 160 stentless patients and 247 stented patients [14], while Luciani et al. analyzed 292 stented patients and 376 stentless patients [15].

We have also shown equipoise in most 30-day clinical outcomes between stented and stentless valves. As shown in Table 2, the incidence of atrial fibrillation, myocardial infarction, stroke, and renal failure as well as the length of hospital stay, ventilation time, and the total length of stay in the intensive care unit were statistically similar between the two valve types. While studies have found that stented and stentless valves have comparable clinical outcomes and hemodynamic profiles such as LVM regression, EOA and transvalvular gradients [8, 10–13], few studies have explicitly reported clinical outcome comparisons similar to our study.

We had a significantly higher incidence of bleeding requiring reoperation in stented valves. Contrary to our findings, prior studies have shown no significant difference in postoperative bleeding between stented and stentless valves [6, 14, 15]. Ali et al. found a 5% incidence of bleeding in a stented cohort vs. 6% in a stentless cohort [10]. We believe that most of the bleeding was coming from the aortotomy in the noncoronary sinus. The stentless valve is placed via a subcoronoary technique that leaves the noncoronary sinus intact, and it may be easier to visualize the aortotomy and thus control any suture line bleeding.

The incidence of stroke was significantly higher in our stentless cohort. This finding is unexpected, and the difference between cohorts has not been shown in prior literature. While the incidence of stroke in our stentless group (2.9%) was similar to that shown in prior studies, the incidence in the stented cohort was lower (0.9%). Borger et al. found a 2% incidence of stroke in both groups [12]. Similarly, Cheng et al. showed a 3.6% incidence of stroke in the stentless group and 4.0% in the stented group [6]. It is possible that the long suture lines required for a stentless valve could lead to increased thrombus formation. Further study in a large cohort is necessary to elucidate the contributing factors to the increased incidence of stroke in stentless valve replacement.

Although prior studies have reached conflicting conclusions regarding the hemodynamic superiority of stentless versus stented valves, our results indicate that the 30-day clinical outcomes and long term survival of the two valves do not differ significantly regardless of any hemodynamic differences. However, there may be instances for which one valve may be preferred over the other. Multiple studies have suggested that stentless valves produce greater improvement in left ventricular function in patients with a small aortic root or left ventricular impairment [3, 9, 10]. Patients with calcification in the sinuses may not be able to undergo stentless implantation because of inability to perform the suture line under the coronaries.

Furthermore, a study by Kunadian et al. found that stentless valves require a more complex surgery with an average cross clamp time increase of 23 min and a 29 min longer bypass time [7]. Dunning et al. also found that the bypass and cross-clamp times were 10 min longer in stentless patients [4]. Our results failed to show any difference in outcomes despite the potentially longer cross clamp and bypass times. Finally, stentless valves are theorized to have a higher durability due to a lack of stress at the stent site, as mentioned above [2]. However, Forcillo et al. have shown that the Carpentier-Edwards pericardial valve retains excellent durability and a low incidence of valve related complications after 25 years of experience with the valve [18]. This finding complements our study by suggesting that improvements in stented valves have led to similar durability and outcomes with stentless valves.

Limitations to this study include its retrospective nature. However, this is the largest propensity matched study in the literature comparing these bioprosthesis and the large number of patients at risk increases the validity of the long term data. Another limitation to this study is the lack of hemodynamic performance data such as effective orifice area, peak/mean gradients, and LVM regression. While we have survival data we did not assess the quality of life in survivors. The causes of death, valve dynamics, and reoperations were also not able to be determined. Choice of valve was at the discretion of the surgeon and this bias could not be controlled. With any propensity matched study the two groups could have confounders that were not able to be identified by the 13 matching parameters chosen.

## Conclusions

In conclusion, stented and stentless valves offer similar clinical outcomes and good long term survival. Stented valves exhibited a decrease in the incidence of stroke, and an increase in postoperative bleeding that required re-exploration three times as often as with stentless valves. Both valves are excellent choice for AVR and have equivalent outcomes.

## Abbreviations

AI: Aortic insufficiency
AS: Aortic stenosis
AVR: Aortic valve replacement
CABG: Coronary artery bypass graft
EOA: Effective orifice area
ICU: Intensive care unit
IRB: Institutional review board
LOS: Length of stay
LVM: Left ventricular mass
MI: Myocardial infarction
NYHA: New York Heart Association
PVD: Peripheral vascular disease
SD: Standard deviation
STS: Society of Thoracic Surgeons
